# Unprovoked Deep Vein Thrombosis as a Manifestation of Cushing's Disease

**DOI:** 10.7759/cureus.104148

**Published:** 2026-02-23

**Authors:** Sharini Venugopal, Maunank Patel

**Affiliations:** 1 Endocrinology, MedStar Shah Medical Group, J. Patrick Jarboe Medical Center, California, USA; 2 Internal Medicine, MedStar Shah Medical Group, J. Patrick Jarboe Medical Center, California, USA

**Keywords:** acth secreting macroadenoma, cortisol hypersecretion, cushing’s disease, deep vein thrombosis (dvt), glucocorticoid withdrawal, hypertension, obesity and overweight, pituitary adenoma surgical treatment, pituitary disorder, recurrent vte

## Abstract

Cushing's disease is a rare condition, and patients have a higher risk of thromboembolism, specifically venous events. We present a case of a middle-aged woman who underwent sleeve surgery many years ago and slowly regained weight. She had two episodes of unprovoked deep vein thrombosis (DVT), and no risk factors were identified at the time of these events. She presented 10 years later with easy bruising, myopathy, gradual weight gain, and insomnia, and was found to have ACTH-dependent pituitary Cushing’s. She successfully underwent endoscopic resection of her pituitary macroadenoma. She had worsening myalgia and myopathy immediately after surgery and is now recovering from glucocorticoid withdrawal syndrome after having untreated Cushing's disease for many years. Her insomnia has resolved almost completely, she has lost 60 pounds, her hypertension has resolved completely, and now she is off medications and remains in remission after 22 months. We conclude that Cushing’s disease should be viewed as a risk factor for unprovoked venous thromboembolism and screened for, especially in obese patients.

## Introduction

Cushing’s disease is a rare disease with an incidence of 6.2 to 7.6 per million person-years in the United States [1]. Cushing's disease patients have a higher risk of thromboembolism, specifically venous events. Cardiovascular deaths and pulmonary embolism can contribute to more than 50% of the mortality from Cushing's disease [2]. Many studies have shown that this could be a result of a combination of increased coagulation factors and a decline in the fibrinolysis pathway [2]. The clinical presentation of the disease can vary widely and can range from mild disease, with cyclical symptoms, to severe disease [2]. Herein, we present a case of a woman who presented with a multitude of symptoms, but her unprovoked deep vein thrombosis (DVT) was an important event in her medical history. She was diagnosed with Cushing’s disease many years later.

## Case presentation

A woman in her mid-50s with a medical history significant for multinodular goiter, adenocarcinoma of the colon, fibromyalgia, obesity status post sleeve surgery, hypertension, two episodes of unprovoked DVT, and sleep apnea presented for the evaluation of thyroid nodules as well as a multitude of symptoms. During the visit, her most concerning symptoms were weakness in her thighs and muscles, body aches, easy bruising, and insomnia. She had also undergone spine surgery for pain and was receiving cortisone shots earlier, with her last injection received more than a year ago. On further probing her DVT history, she stated that this was unprovoked, and she had two episodes of these and had an inferior vena cava filter placed.

On examination, she was obese with a BMI of 34, had a buffalo hump, and no moon facies or violaceous stretch marks were seen. Her muscle strength was graded 5 out of 5 bilaterally, and lungs and heart sounds were normal. A dexamethasone suppression test showed a cortisol level of 13 ug/dL (normal, <1.8 ug/dL) with a dexamethasone level of 395 ng/dL (140-295 ng/dL). The baseline adrenocorticotropic hormone (ACTH) level determined using the Roche assay was 69.8 pg/mL (7.2-63.3 pg/mL). Complete blood count and comprehensive metabolic panel were unremarkable. The urinary cortisol level was 140 ug per 24 hours (6-42 ug/24 hours), with creatinine at 1071 mg/24 hours; salivary cortisol was at 0.219 ug/dL and 0.132 ug/dL (<0.010-0.090 ug/dL) on two consecutive nights. She had three out of three tests positive for hypercortisolism, and we proceeded with an MRI given the high ACTH (Table 1).

**Table 1:** Laboratory results ACTH: adrenocorticotropic hormone

Test	Value	Reference Range
Low-dose dexamethasone suppression test, cortisol	13 ug/dL	<1.8 ug/dL
Dexamethasone level	395 ng/dL	140-295 ng/dL
ACTH Roche assay	69.8 pg/mL	7.2-63.3 pg/mL
24-hour urinary cortisol	140 ug per 24 hours	6-42 ug/24 hours
Midnight salivary cortisol	0.219 ug/dL	<0.010-0.090 ug/dL
Midnight salivary cortisol	0.132 ug/dL	<0.010-0.090 ug/dL
High-dose dexamethasone suppression test, cortisol pre-dexamethasone	24 ug/dL	8-19 ug/dL
Cortisol post-dexamethasone	1.6 ug/dL (>50% decline)	<1.8 ug/dL
ACTH pre-dexamethasone	65.4 pg/mL	7.2-63.3 pg/mL
ACTH post-dexamethasone	17.6 pg/mL (>50% decline)	7.2 to 63.3 pg/mL
Alpha subunit	1.2 ng/mL	<2.34 ng/mL
Estradiol	16 pg/mL	<6-54.7 pg/mL
Follicle-stimulating hormone	83.2 mIU/mL	Post-menopausal, 25.8-134.8 mIU/mL
Insulin-like growth factor 1	216 ng/mL	53-287 ng/mL
Prolactin 1:100	19 ng/mL	3.6-25.2 ng/mL
TSH	1.021 uIU/mL	0.400-6.000 uIU/mL
Free T4	0.81 ng/dL	0.71-1.50 ng/dL

**Figure 1:**
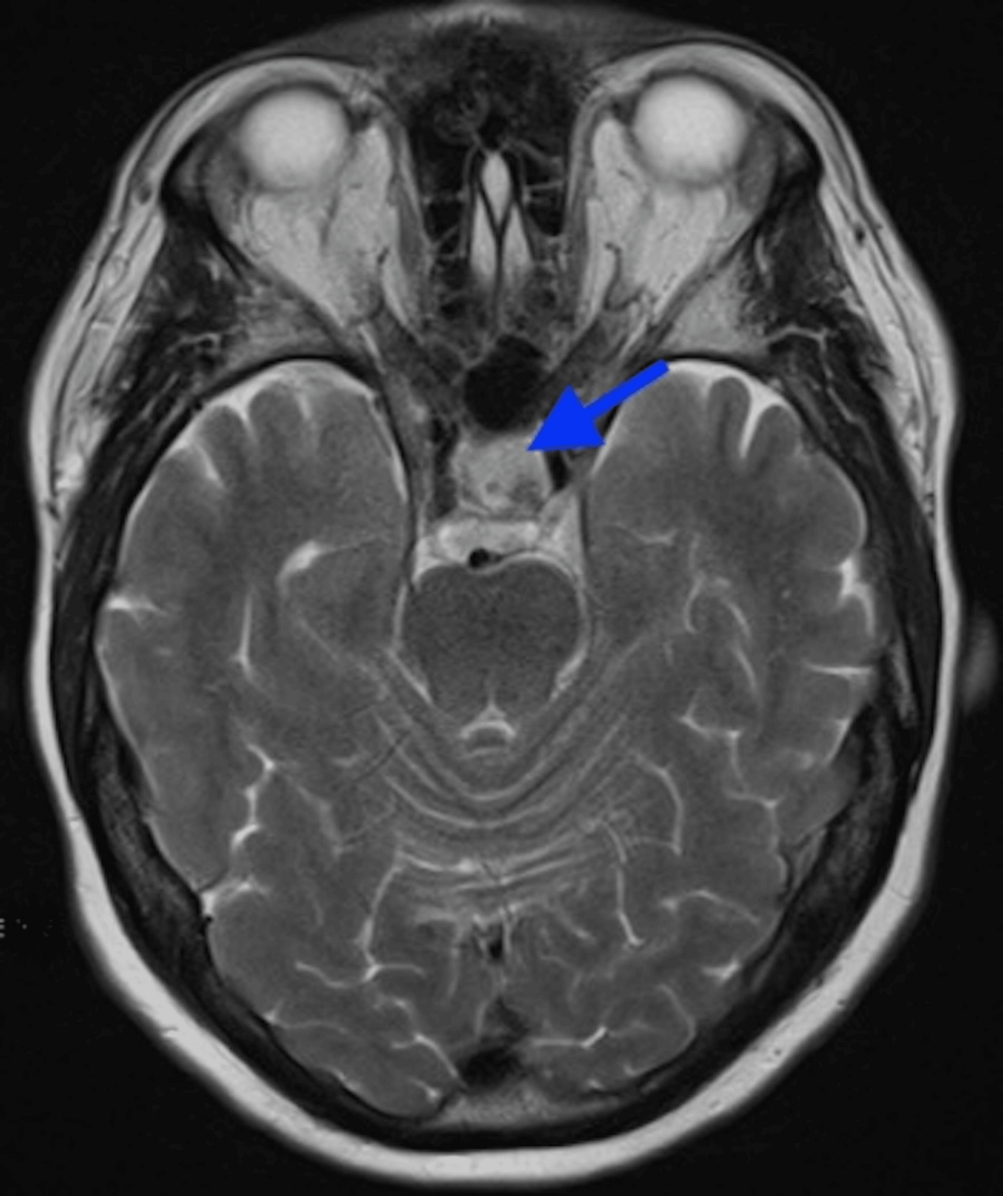
Brain MRI with pituitary protocol showed a new macroadenoma in the left pituitary moiety (marked by an arrow), measuring up to 1 cm

An MRI of the brain using a pituitary protocol showed a new macroadenoma in the left pituitary moiety measuring up to 1 cm (Figure 1). No cavernous sinus extension was seen, and the optic chiasm was intact.

A high-dose dexamethasone test revealed an ACTH decline from 65.4 pg/mL to 17.6 pg/mL and a cortisol decline from 24 ug/dL to 1.6 ug/dL. The baseline alpha subunit level was 1.2 ng/mL (normal, <2.34 ng/mL), estradiol was at 16 pg/mL (normal, <6 to 54.7 pg/mL), FSH was at 83.2 mIU/mL (post-menopausal, 25.8-134.8 mIU/mL), insulin-like growth factor 1 (IGF-1) at 216 ng/mL (normal, 53-287 ng/mL) and prolactin 1:100 was at 19 ng/mL (3.6-25.2 ng/mL) (Table 1).

She successfully underwent endoscopic resection of the pituitary tumor. Pathology revealed a corticotroph pituitary neuroendocrine tumor (PitNET). Sections of the pituitary macroadenoma revealed a small fragment of fibrotic and compressed adenohypophysis and a second fragment of fibrous tissue involved by the corticotroph PitNET. Immunohistochemical stains for PIT1 (pituitary-specific transcription factor 1), TPIT (T-box transcription factor), and SF-1 (steroidogenic factor 1) each highlighted scattered adenohypophyseal cells, while TPIT and ACTH highlighted tumor cells and the dense fibrous tissue fragment, supporting involvement by the corticotroph PitNET. The Ki-67 proliferation index (a marker of proliferation) was approximately 3%-4%.

The patient was started on dexamethasone preoperatively, and hence, postoperative cortisol was 0.7. She was discharged on hydrocortisone 30 mg in the morning and 20 mg in the afternoon, and we slowly weaned her to hydrocortisone 10 mg in the morning and 5 mg in the afternoon. The patient experienced cortisone withdrawal with body aches immediately after surgery, and she lost close to 60 pounds after surgery. She is currently 22 months post-surgery, and her most recent ACTH stimulation test went up to 12 ug/dL (normal cortisol level for patients with full hypothalamic pituitary axis recovery being 18 ug/dL, to be able to wean off steroids). She still has not recovered completely since her cortisol peaked at only 12 ug/dL, and continues to receive hydrocortisone 10/5 mg daily.

## Discussion

Patients with Cushing’s disease have a higher risk of thromboembolism [2]. Thromboembolism contributes to the higher mortality rates in this population in the form of stroke, ischemic cardiac disease, and pulmonary embolism [3]. This is thought to be due to increased metabolic function due to endothelin cells, leading to increased thrombin formation [4]. A retrospective study with 208 patients undergoing pituitary surgery or adrenalectomy showed an approximate risk of 20%, with most events occurring between 30 and 60 days postoperatively [4,5]. Most studies have shown an increased risk of VTE in Cushing’s disease, anywhere from 10% to 20% [5,6].

A Denmark-based study looked into mortality in Cushing’s disease and found that mortality was twice as high in these patients. They had an increased risk of VTE, myocardial infarction (MI), stroke, fractures, peptic ulcer, and infections. Interestingly, the mortality risk and MI risk remained elevated despite surgery, while the VTE risk was high in the initial perioperative period, according to this study. This study also concluded that the rates of VTE were similar in adrenal as well as pituitary Cushing’s disease [7].

Interestingly, studies have shown that the degree of urinary free cortisol elevation does not correlate with comorbidities. Hence, the clinical features may not always correlate with the degree of hypercortisolism [8]. Another study concluded that no linear relationship was found correlating the severity of endogenous hypercortisolism with the number of thrombotic events or laboratory metrics of coagulation [9]. The odds of VTE in patients undergoing transsphenoidal surgery or adrenalectomy were lower than in patients undergoing orthopedic surgery for hip fracture repair, and the VTE risk was intermediate when compared between the general population and orthopedic surgery patients [9]. The same study concluded that the odds ratio for VTE in Cushing's syndrome (CS) patients was 17.8 compared to the healthy population [9]. Most of the CS patients in this study were middle-aged women, just like our patient.

A large European multicenter study showed that preoperative medical treatment of hypercortisolism does not eliminate the TE risk [10]. Some studies have shown that a decrease in cortisol after surgery can increase VTE risk paradoxically, possibly from the withdrawal of the anti-inflammatory effects of cortisol [11]. Some studies showed that medical therapy did not change the clotting factors, but no patient developed a thrombotic episode during treatment with pasireotide [9,12].

von Willebrand factor (vWF) mediates platelet adhesion and stabilizes Factor VIII (FVIII), which is a risk factor for thrombus formation [13]. The hypercoagulable state results from a combination of increased thrombogenic factors and impaired fibrinolysis [14]. Prothrombotic factors such as vWF, fibrinogen, and FVIII are found to be increased, while others (activated partial thromboplastin time, or APTT) are decreased in CS patients. Also, anti-thrombotic markers (protein-C and protein-S) are increased [9]. In addition, these patients have abdominal obesity, which is associated with increased levels of Factor VII, vWF, and plasminogen activator inhibitor-1 [15].

A 2022 survey of the current clinical practices for thromboprophylaxis management in patients with CS across reference centers of the European Reference Network on Rare Endocrine Conditions showed that the VTE prophylaxis varied between different centers, and most centers did not follow any one protocol [16]. The majority of the centers reported previous history of VTE or prolonged immobilization or degree of hypercortisolism as risk factors to initiate therapy [16]. There was one study that designed a tool in 2016 to estimate the risk of VTE in CS patients [17]. The risk factors were age ≥69 years, reduced mobility, acute severe infections, previous cardiovascular events, midnight plasma cortisol level >3.15 times the normal, and shortened APTT [17].

## Conclusions

Cushing's disease is a risk factor for venous thromboembolism. Currently, an individualized plan is recommended for thromboprophylaxis based on patient risk factors such as the use of estrogen, obesity, etc.; VTE prophylaxis should be discontinued before transsphenoidal adenomectomy to reduce the risk of intraoperative bleeding. We need larger prospective studies to look into the duration, timing, and preferred anticoagulation in these patients perioperatively, and also to add CS as a risk factor in VTE guidelines.
